# A glasses-type wearable device for monitoring the patterns of food intake and facial activity

**DOI:** 10.1038/srep41690

**Published:** 2017-01-30

**Authors:** Jungman Chung, Jungmin Chung, Wonjun Oh, Yongkyu Yoo, Won Gu Lee, Hyunwoo Bang

**Affiliations:** 1 School of Mechanical and Aerospace Engineering, Seoul National University, Seoul 08826, Republic of Korea; 2 Graduate School of Convergence Science and Technology, Seoul National University, Suwon 16229, Republic of Korea; 3 Department of Mechanical Engineering, Kyung Hee University, Yongin 17104, Republic of Korea; 4 Envisible, Inc., Seoul 06127, Republic of Korea

## Abstract

Here we present a new method for automatic and objective monitoring of ingestive behaviors in comparison with other facial activities through load cells embedded in a pair of glasses, named GlasSense. Typically, activated by subtle contraction and relaxation of a temporalis muscle, there is a cyclic movement of the temporomandibular joint during mastication. However, such muscular signals are, in general, too weak to sense without amplification or an electromyographic analysis. To detect these oscillatory facial signals without any use of obtrusive device, we incorporated a load cell into each hinge which was used as a lever mechanism on both sides of the glasses. Thus, the signal measured at the load cells can detect the force amplified mechanically by the hinge. We demonstrated a proof-of-concept validation of the amplification by differentiating the force signals between the hinge and the temple. A pattern recognition was applied to extract statistical features and classify featured behavioral patterns, such as natural head movement, chewing, talking, and wink. The overall results showed that the average F_1_ score of the classification was about 94.0% and the accuracy above 89%. We believe this approach will be helpful for designing a non-intrusive and un-obtrusive eyewear-based ingestive behavior monitoring system.

Maintaining energy balance in the human body is a vital condition, as abnormal or excessive energy accumulation is the central cause of obesity^1^. In 2014, according to an announcement by the WHO, the obesity rate has more than doubled since 1980; further, 39% of adults aged 18 years or older are overweight^2^. These figures indicate that energy imbalance is a worldwide prevalent epidemic; this condition is serious as it can promote many medical complications, such as stroke, heart disease, and cancer^3^. Although its etiology is still incompletely understood, the drastically increasing rate of recent years in modern societies suggests that a behavioral etiology is considered as a significant factor as a biological one^4^; therefore, reduced activity and changes in eating patterns must be monitored continuously over a long time span. Obviously, this behavioral monitoring can also help normal people, who are not suffering from obesity or other eating disorders, in maintaining a healthy life.

There have been many discussions on the monitoring of physical activity^5^,^6^,^7^,^8^, and a variety of commercial products have already been introduced at the consumer level. Monitoring of ingestive behavior (MIB), however, has been less studied in practice due to the difficulty of direct and objective measurement of food intake. There have been different approaches in the MIB such as acoustical approaches based on chewing or swallowing sounds^9^,^10^, morphological approaches sensing deformation of the epidermis^11^,^12^, behavioral approaches using a proximity sensor or an inertial measurement unit (IMU)^13^,^14^, and electrometric approaches analyzing facial muscle activity^15^,^16^. These approaches, however, share common limitations in that they are obtrusive to the eye and intrusive to use in daily life; therefore, we introduce a non-intrusive and un-obtrusive method of direct and objective monitoring of ingestive behavior employing the use of wearable devices.

This study was motivated by two ideas: (i) measurement of temporalis muscle activity due to its role as a masticatory muscle during ingestive behavior; and (ii) the natural lever mechanism of a pair of glasses, which pass through the temporalis epidermis when equipped. To explain the first idea, contraction and relaxation of the temporalis muscle result in elevation, retraction, and side-to-side grinding movements of the mandible, or lower jawbone, during the mastication cycle^17^. This muscle activity results in approximate 1.2 *mm* changes of the muscle thickness, with a lower deviation compared with that of the masseter and sternocleidomastoid muscles for adults without temporomandibular disorder^18^. Based on this background, this study utilized oscillatory patterns of the thickness of the temporalis muscle for the MIB. Here, we focused on the second idea and employed glasses that are fastened by friction due to a compressive force at the contact area between the temples of the glasses and the temporalis epidermis on both sides of the head. In other words, we can monitor the temporalis muscle activity by measuring the force exerted onto the temple areas of the glasses. This force signal, however, has several weaknesses; (a) it is too weak to be detected directly from the contact area, (b) it is distributed over the contact area, (c) both location and form factors of the contact area differ from individual to individual, and (d) direct contact with the epidermis exposes the sensor to possible damage from perspiration and rubbing. To resolve these problems, we proposed the use of a mechanical advantage created by the natural lever mechanism of the glasses. By measuring the force on the hinge, where the temple contacts the headpiece, it becomes much easier to detect the temporalis muscle activity during ingestive behavior. This solution provides the following advantages: (a) the force is amplified by the laws of a lever, (b) the force is concentrated on the small contact area between the temple and the headpiece, (c) the uniform form factor accommodates for variety in individuals, and (d) the sensor avoids damage from direct contact with the epidermis. The graphical description of these features and advantages is illustrated in Fig. 1.

Recently, there has been more practical approaches to recognize chewing events with utilizing the temporalis muscle activity and a glassed-type wearable device. Farooq and Sazonov^19^ attached a piezoelectric sensor onto temporalis epidermis to monitor chewing cycles and collected data through a Bluetooth module embedded in a pair of glasses. So they could monitor the eating behavior even in walking condition. Zhang, Bernhart, and Amft^20^ used electromyography (EMG) electrodes in a pair of 3D-printed glasses. They designed the suitable placements and the type of the EMG electrodes within the glasses. However, they had common limitations that the sensor could easily be damaged or influenced by the perspiration or hairs between the skin and the sensor. Our approaches effectively solved this problem by taking advantage of kinematics of the glasses itself. Furthermore, utilizing the two sensors on both sides could differentiate left-and-right chewing and wink events.

In this study, we present a new method for the MIB utilizing the natural lever mechanism of a pair of glasses, named GlasSense. To verity the amplification on the hinge, we conducted an experiment on comparing the force directly exerted on the temple area and its transmitted force on the hinge. In fact, this amplification principle mimics that of sound in the inner ear: the vibration of air (temporalis muscle activity) is amplified by the lever mechanism through the auditory ossicle (temple); and the transmitted force is concentrated on the small stapes footplate area (hinge), compared with the large eardrum area. For practical application, we analyzed left-and-right chewing behaviors and distinguished these from the other facial activities, such as natural head movement, talking and wink. Therefore six distinct behavior sets from 10 subjects were collected and labeled into the corresponding set: natural head movement (NHM), left chewing (LC), right chewing (RC), left wink (LW), right wink (RW), and talking (TK). Then, algorithms for signal preprocessing, feature extraction, and supervised machine learning were proposed for the classification of the sets.

## Results

### Validation of Force Amplification

We set a separate experimental apparatus to conduct an objective experiment on the comparison of the forces between the hinge and the temple for the validation of the mechanical amplification. Figure 2 shows these settings which were composed of a fixed stage equipped with a linear sub-stage, a micrometer dial gauge, and the glasses. As displacement of the linear sub-stage was changing, the force signal from the hinge, *F*_*hinge*_, showed a large rate of increase in magnitude compared with that from the temple, *F*_*temple*_ (Fig. 3a). From linear regressions with the least square method which minimizes the sum of squared residuals, we obtained lines-of-best-fit of *F*_*hinge*_ and *F*_*temple*_. The both measured force signals showed high linearities with coefficients of determination of *R*^*2*^ = 0.998 for the hinge and *R*^*2*^ = 0.993 for the temple. The regressions were significant with p-values of *p* = 1.53e-121 for the hinge and *p* = 2.43e-95 for the temple. The slopes calculated from the regression coefficients of the lines-of-best-fit also showed the large rate of increase in magnitude from the hinge (slope = 3.31 *N/mm*) compared with from the temple (slope = 0.44 *N/mm*).

Using *F*_*temple*_ and *F*_*hinge*_ signals as x- and y-values, respectively, we obtained the experimental amplification factor from the regression coefficients (slope) of the line-of-best-fit (*R*^*2*^ = 0.997 and *p* = 2.62e-110); the result showed that the slope was about 7.57, which was almost the same as the theoretical amplification factor, 7.67 (Fig. 3b). The theoretical-amplified force is calculated by the moment equilibrium of the temple-hinge joint-head piece system as follows:


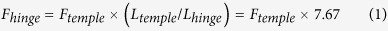


### Detection of Temporalis Muscle Activity

The purpose of this study was to design a pair of glasses and define a classifier model that consistently sense temporalis muscle activity regardless of intra- and inter-individual variability. Thus, we collected and analyzed the data from 10-subject user study to define only one SVM classifier corresponding to all subjects. We recorded force signals per every consecutive window of 3-second frame. This window size was chosen from the fact that chewing frequency mainly ranges from 0.94 *Hz* (5^th^ percentile) and 2.17 *Hz* (95^th^ percentile)^21^. So a 3-second single window could contain multiple chewing activities. In order to reflect the variety of food properties, the chewing sets were conducted for three different food textures: bread (sliced white breads and croissants); potato chip (Lay’s); and jelly (Jelly Belly). Each item differentiates its texture from a distinct hardness, crispiness, and tackiness. For example, the bread represents the soft, the potato chip does the hard and crispy, and the jelly does the soft and tacky texture.

In order to extract appropriate features for the classification, we used statistical features from temporal and spectral domains of a window (3-second recording frame). The entire feature list can be found in Supplementary Table S1. Total number of 84 features was used to build a feature vector with a label ranged from 1 to 6 depending on its behavioral set.

Figure 4 shows the procedure and the results to determine the SVM classifier model with the optimal parameters, (*C, γ*). A grid-search procedure was adopted to find the model of the best accuracy. The exponentially growing pairs of (*C, γ*) were examined by a two-step grid-search procedures, coarse (left) and fine (right) grid, respectively. The *γ* had the local optimal range from 2^−2^ to 2^2^ while the *C* showed saturated values when it increases more than 2^10^. The fine grid was chosen from this basis. The results show that the maximum accuracy was about 94.6% at (*C, γ*) = (16, 1). In order to avoid overfitting problems and remove the inter-individual variability, the training procedure was performed with a 10-fold leave-one-out cross-validation scheme for each pair of (*C, γ*). It divided the entire set into 10-part subsets in order to each subset corresponds to each subject. So, it tested one subject(subset) sequentially while the remaining subjects(subsets) were trained.

Table 1 shows the classification results on the entire behavior set through a confusion matrix, which represents both the predicted behaviors and the actual behaviors with precisions, recalls, and F_1_ scores. The diagonal elements represent the true positives (TP) of each class, whereas the row and column elements on the off-diagonals represent false positives (FP) and false negatives (FN), respectively. Thus, the precisions (outcome accuracy), recalls (condition accuracy) were obtained from


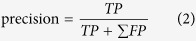



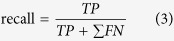


The F_1_ score is a measure of the accuracy which considers both the precision and the recall. It is computed from the harmonic mean of the precision and the recall with as follows:


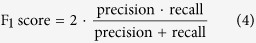


The F_1_ scores of the NHM, LW, RW and TK showed the high prediction results (>93%) while those of the LC and RC showed the relatively lower prediction results (>89%). Table 1 shows these results were mainly from the misclassification between the LC and RC; in other words, classification between chewing and the other behaviors showed sufficient prediction performance. It was also observed that the misclassification significantly occurred between the NHM and the TK. The TK showed slightly large amount of misclassification from all of the other behaviors. The average F_1_ score was obtained as 94.0% from the arithmetic average of the F_1_ scores.

## Discussion

In this study, we presented a novel method to sense temporalis muscle activity through load-cell-embedded-glasses, GlasSense. Utilizing the natural lever mechanism between the temple and the head piece, we obtained an amplified and concentrated force on the hinge. This mechanical amplification was verified by comparing the force between the temple and the hinge (Fig. 3). The experimental amplification factor, 7.57, showed almost the same value as the theoretical one, 7.67. It is expected that the slight decrease in the experimental value was due to measurement errors and other reaction forces in the real world, such as friction of the hinge joint. With the exception of such factors, the amplification factor is purely influenced by the geometric properties, *L*_*temple*_ and *L*_*hinge*_, according to the moment equilibrium eq. (1). So, we can increase the amplification factor by increasing the proportion of *L*_*temple*_ to the *L*_*hinge*_ as much as possible.

When the exerted force on the temple was smaller than 0.5 *N*, a large deviation of the data from the line-of-best-fit was observed as shown in Fig. 3b. We assume two possible reasons: the contact and the exerted force were insufficient to measure the meaningful data in this condition (<0.5 *N*); and weight of the temple and the electronic wires primarily affected the load cell on the hinge when the applied force was extremely small. When enough force greater than 0.5 *N* was exerted on the temple, the experimental results were in a good agreement with simulation results for amplification factors. In general wearing condition, this gravitational and contactless factors can be discarded because the glasses is equipped with the perpendicular direction to the gravity, and the sensors have pre-loaded compressive force enough to support the glasses against the gravity.

In practical wearing situation, the difference in the contact area between the temple and the hinge also affect the amplification factor. The contact area of the temple is larger than that of the hinge and differ from individual to individual. The transmitted force to hinge, meanwhile, can be concentrated and accommodate a variety of individual’s form factors. With the consideration of the force concentration, the amplification factor is expected to become much larger than the experimental result.

We applied machine learning algorithms to classify patterns of the temporalis muscle activity with regard to the chewing and the other facial behaviors. From the left-and-right measurement of the force of the glasses, the LC and the RC could be classified with the F_1_ scores greater than 89%. This value was relatively lower than the average F_1_ score, 94.0%. The main reason of it was due to the misclassification between the LC and the RC (more than 10% of the true positive), whereas the classification of the chewing from the other activity showed high prediction results. It was assumed that the precise user study with one-sided mastication was hard to be controlled during all through the experiment; thus the correlation features between left-and-right sides could affect the results incorrectly. Nevertheless, these results suggest that the chewing activity can be classified from the other temporalis muscle activities showing the distinct and unique patterns.

It was observed that the misclassification also occurred between the NHM and the TK. The NHM represented the practical noise from the natural head movement in sedentary condition. Users were allowed to stay at rest, turn their head around, use a smart phone freely, or do a light stretching without chewing, talking, or wink which may affect the temporalis muscle activity. Figure 5 shows the temporal signals of each behavior. We can confirm that these behavioral noises were definitely discriminated from the chewing and wink event in terms of shapes and amplitude, while the talking and the NHM revealed much fewer distinction between them. The talking was the most unpredictable set because it was accompanied by complex motions with the jaw, head, and even hands.

We are continuing this research in order to enhance practical usability in daily life. There are more dynamic physical behaviors, such as head shaking, walking, and running, which can affect the measured force signal from the hinge. In order to examine the effects of those behaviors, an extended user study will be conducted. Real-time algorithms and wireless capability are also being tested with consideration of unintended environmental noise such as walking. This can be achieved by adding a MEMS accelerometer sensor to the device to cancel the force due to the body movement. From this sensor fusion technique, the monitoring of both the energy intake and energy expenditure can be tracked in daily life.

Our approach has another strength on the sensing methodology because it utilizes the indirect contact with skin. If the sensor is attached or contact with the epidermis, it is prone to be damaged or removed from the body. We expect that the non-intrusive and contactless sensors in the form of the wearable glasses can achieve a robust monitoring of ingestive behaviors with a higher accuracy through these investigations.

Here, we also want to discuss potential of our device as glassware wearable capable of recognizing facial patterns in a hands-free controller format. Recently, Google glass-based wearable device has been developed to control voice recognition commands into their biomedical applications^22^. Their approach seems very creative and helpful for people who may require hands-free behaviors and motions in a controlled manner. In this sense, this study can give us the potential to supplement the lack of input methods in AR or VR applications without disrupting other activities. We further believe that our approach can be an innovative wearable device for monitoring and controlling a set of facial behaviors such as chewing and talking in a daily lifecare format.

## Methods

In this study, all the experiments were performed by simply wearing the glasses. All the signals were recorded by measuring the signal from load cells inserted in the glasses. All methods were carried out noninvasively like a common situation of wearing a pair of glasses as usual. All experimental protocols were not related to *in vivo/in vitro* human studies. No drug and blood samples were used for the experiments. Informed consent was obtained from all subjects of experiments.

### Experiment on Force Amplification

We designed and 3D-printed frames of a pair of glasses and embedded two ball-type load cells (FSS1500NSB, Honeywell, USA) in one of the temples. One load cell was placed between a gap in the hinge where the temple contacts the headpiece, and the other was on an assumed contact point with the epidermis, which was 69 *mm* from the hinge joint in this experiment (Fig. 2). The device was fixed to a stage, which had a linearly movable sub-stage to contact the load cell of the temple and a micrometer dial gauge to measure the displacement of the temple. We applied the force to both load cells by moving the sub-stage with a 50 *μm* resolution.

### Detection of Temporalis Muscle Activity

**Figure 1:**
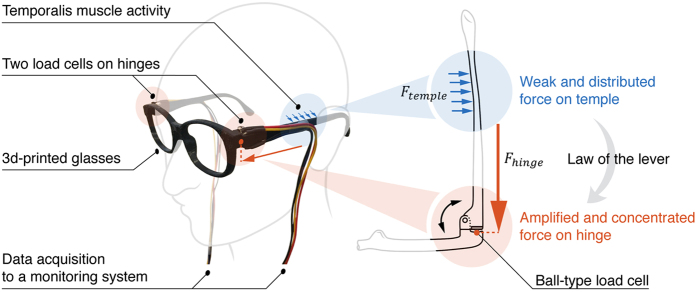
Schematic diagrams of GlasSense, glasses that sense weak oscillatory patterns of the temporalis muscle (left), and its working principle (right). Two ball-typed load cells are embedded in hinges on both sides of a pair of 3D-printed glasses. The acquired force signals are transmitted to a monitoring system to detect temporalis muscle activity during ingestive behavior and other referential activities, such as natural head movement, talking, and wink. When activated by the lever mechanism with a very small contact area on the hinge, the load cells can measure an amplified and concentrated compressive force. This solution, placing the load cells on hinges and utilizing the natural lever mechanism of the glasses, has other advantages such as accommodating a variety of individuals with a uniform form factor and avoidance of damage from direct contact with the epidermis.

**Figure 2:**
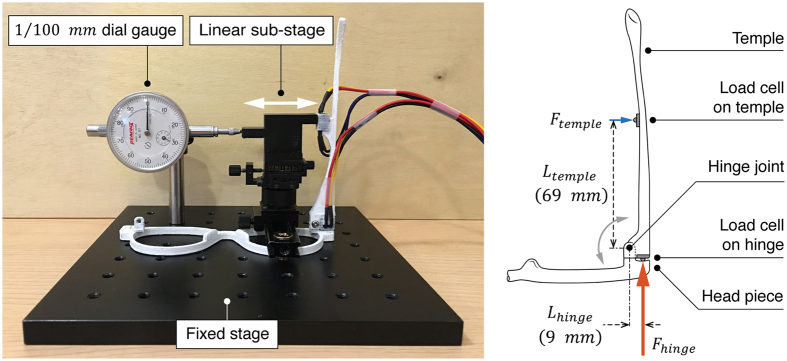
Experimental apparatus (left) and dimensions (right) for the validation of the mechanical amplification caused by the lever mechanism between the force on the temple, *F*_*temple*_, and the force on the hinge, *F*_*hinge*_. The 3D-printed glasses were fixed to a stage equipped with a micrometer dial gauge and a linear sub-stage to contact the temple. In order to reflect general wear conditions, the load cell on the temple was placed at an assumed contact point with the epidermis, 69 *mm* away from the hinge joint in this experiment. We applied the force to the load cells by moving the linear sub-stage with a 50 *μm* resolution. From the moment equilibrium, the theoretical-amplified force on the hinge is calculated by eq. (1).

**Figure 3:**
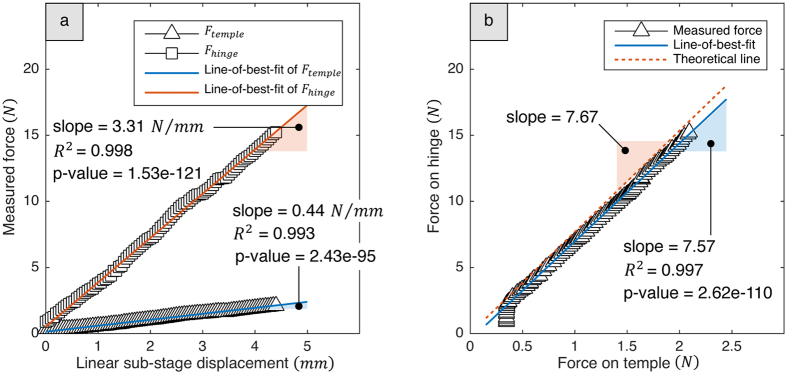
Experimental results for the validation of mechanical amplification. (**a**) Scatter plots of the two force signals and their lines-of-best-fit from linear regressions. High linearities and significant result were obtained from the *F*_*hinge*_ (*R*^*2*^ = 0.998 and *p* = 1.53e-121) and *F*_*temple*_ (*R*^*2*^ = 0.993 and *p* = 2.43e-95) data. (**b**) Comparison of the experimental amplification factor with the theoretical one. The *F*_*hinge*_ and *F*_*temple*_ were used as x- and y-values respectively to define the amplification factor from a slope of a line-of-best-fit (*R*^*2*^ = 0.997 and *p* = 2.62e-110). It is confirmed that the mechanical amplification was made at the hinge with the amplification factor of 7.57.

**Figure 4:**
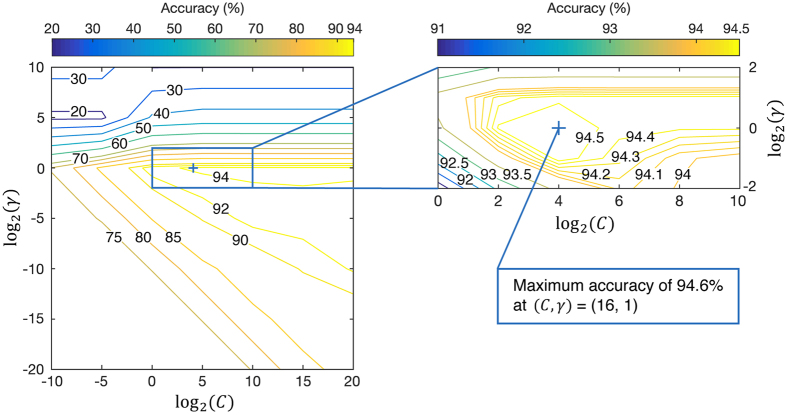
Accuracy results of various pairs of (*C, γ*) values to define a model of the SVM classifier. The SVM with an RBF kernel has two model parameters: the penalty parameter, C, and the kernel parameter, *γ*. The pair of (*C, γ*) with the best accuracy was obtained from both coarse (left) and fine (right) grid-search procedures of exponentially growing pairs of (*C, γ*) values.

**Figure 5:**
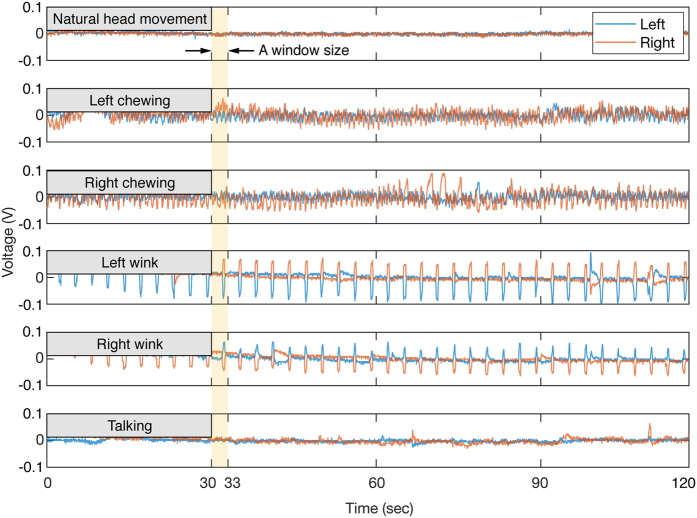
Samples of left-and-right-measured force signals of a subject for six distinct behavior sets. A measurement unit of the signals was voltage which was subtracted by the median of it, as a pre-loaded voltage was different from the subject’s head size and wearing conditions. Therefore, it was not used as a feature for classification. Every sample was composed of a 3-second recording time, since it had sufficient information on the entire cycle of the each behavior set.

The main idea of this study was to monitor the temporalis muscle activity using the amplified and concentrated forces on the hinge. In order to collect those signals on both left and right sides, the same ball-type load cells were embedded in the hinges on both sides of the glasses (Fig. 1). When pressed by the lever mechanism with a very small contact area on the hinge, the ball of the sensor can measure an amplified and concentrated compressive force. Using this device, we conducted a user study of 10 subjects (seven males and three females, the average age was 24.9 ± 2.5 (s.d.: standard deviation) years, and the average body mass index (BMI) was 22.29 ± 2.60 (s.d.) *kg/m*^*2*^) for six distinct behavior sets: natural head movement (NHM), left chewing (LC), right chewing (RC), left wink (LW), right wink (RW), and talking (TK). The talking set reflects conversation during a meal in order to distinguish from actual ingestive behaviors. In order to record unbroken temporal data, users read a book at a general voice volume and speed instead of talking with another person. We collected a total number of 20,700 samples, each of which was composed of a 3-second time interval so as to contain the multiple cycles of the behaviors as shown in Fig. 5. The difference in shape of the curves can be observed among the sets as the force was amplified by the mechanical advantage enough to be discriminated.

**Figure 6:**
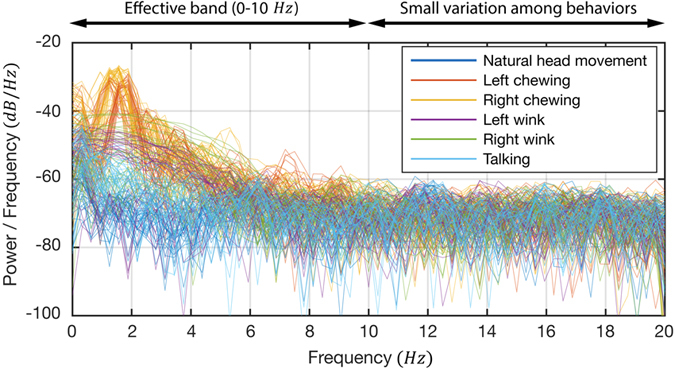
Power spectral density (PSD) functions of the windows for all sets. The unfiltered signals show that values higher than 10 *Hz* have too small variation among the sets to use for classification, while the values lower than 10 *Hz* have large variation. A 5th-order Butterworth low-pass filter (LPF) with a cut-off frequency of 10 *Hz* was applied to the PSD results.

In order to classify the collected samples into the corresponding sets, we applied a series of algorithms for signal preprocessing, feature extraction, and supervised machine learning techniques. First, the Hanning window was applied to each window to reduce the spectral leakage on performing the FFT. A low-pass filter with cut-off frequency of 10 *Hz* was then applied to calculated power spectral density (PSD) functions, because the chewing frequency does not exceed 3 *Hz*^21^. From the Fig. 6, it was confirmed that the frequency higher than 10 *Hz* had too small variation among the sets to use for classification. After that, we extracted statistical features from both temporal and spectral domains. The temporal features were calculated from the filtered force signals and the spectral features from a single-sided spectrum of the FFT. The left and right features were calculated separately except for correlation features such as a correlation coefficient and signal magnitude area. The correlation of the left and right force signals enabled the classification of the LC and RC. These features were then scaled and normalized over the whole feature vectors. After that, an 84-dimensional feature vector and the corresponding label were created for each sample. Finally, this feature vector and label were used to train and predict the class using Support Vector Machine (SVM), a well-known classifier that shows excellent performance in generalization and robustness on supervised machine learning problems^23^. In this study, we used the LibSVM^24^ software package for MATLAB to implement the SVM classifier with a Radial Basis Function (RBF) kernel.

## Additional Information

**How to cite this article**: Chung, J. *et al*. A glasses-type wearable device for monitoring the patterns of food intake and facial activity. *Sci. Rep.* **7**, 41690; doi: 10.1038/srep41690 (2017).

**Publisher's note:** Springer Nature remains neutral with regard to jurisdictional claims in published maps and institutional affiliations.

## Supplementary Material

Supplementary Information

## Figures and Tables

**Table 1:** Confusion matrix for the ingestive behaviors with other facial behaviors.

Predicted Behavior	Actual Behavior						Total	Precision
	aNHM	bLC	cRC	dLW	eRW	fTK		
**NHM**	3291	19	5	1	4	119	3439	95.7%
**LC**	2	3089	332	10	10	15	3458	89.3%
**RC**	1	320	3084	7	8	24	3444	89.6%
**LW**	1	4	8	3394	17	17	3441	98.6%
**RW**	8	4	2	19	3353	32	3418	98.1%
**TK**	147	14	19	19	58	3243	3500	92.7%
**Total**	3450	3450	3450	3450	3450	3450	20700	
**Recall**	95.4%	89.5%	89.4%	98.4%	97.2%	94.0%		
**F**_**1**_ **score**	95.5%	89.4%	89.5%	98.5%	97.6%	93.3%		
**Average F**_**1**_ **score**		94.0%						

^a^NHM: natural head movement.

^b^LC: left chewing.

^c^RC: right chewing.

^d^LW: left wink.

^e^RW: right wink.

^f^TK: talking.
