# Supplementary material for: A glasses-type wearable device for monitoring the patterns of food intake and facial activity
Source: Sci Rep. 2017 Jan 30;7:41690. doi: 10.1038/srep41690 (PMC5278398; doi:10.1038/srep41690)
Supplement: Supplementary Information [file srep41690-s1.doc]

Electronic Supplementary Information (ESI)

A glasses-type wearable device for monitoring the patterns of food intake and facial activity

Jungman Chung^1^, Jungmin Chung^2^, Wonjun Oh^1^, Yongkyu Yoo^3^, Won Gu Lee3[[1]](#footnote-2)†, and Hyunwoo Bang^4†^

*^1^ School of Mechanical and Aerospace Engineering, Seoul National University, Seoul 08826, Republic of Korea*

*^2^ Graduate School of Convergence Science and Technology, Seoul National University, Suwon 16229, Republic of Korea*

*^3^ Department of Mechanical Engineering, Kyung Hee University, Yongin 17104, Republic of Korea*

*^4^ Envisible, Inc., Seoul 06131, Republic of Korea*

Hardware Prototyping

We prototyped a 3D-printed glasses frame to use the lever mechanism of hinge. The headpiece and the temple connected through a bolt to freely rotate around the hinge as a pivot joint. The load cells placed at the hinge of temple contact with a headpiece when a user wears the device (Figure 1), measuring the applied compressive force ranging from 0 gf to 1500 gf (gram-force). The sensor operates with an unamplified and uncompensated Wheatstone bridge circuit and provides stable milli-voltage outputs over the force. The voltage signals from the sensor were amplified using an instrumentation amplifier (INA125P, Texas Instruments, USA). The amplification gain G was chosen as 16 to make the input voltage to a microcontroller ranging from 0 to 5 V. Arduino Uno ATmega328P (Arduino Uno, Sparkfun, USA) sampled the signal with a 40 Hz sampling rate and a 10-bit resolution. The serial communication with USB was used to transmit the acquired signal to the monitoring software on a computer.

Data Collection

The data used for the MIB were collected from 10 subjects (7 males and 3 females); the average age was 24.9 ± 2.5 (s.d.: standard deviation) years; and the average body mass index (BMI) was 22.29 ± 2.60 (s.d.) kg/m^2^. All the subjects were selected not to present any physical and medical difficulties on performing normal food intake and talking. We provided them with customized recording software to automatically follow the experimental procedure.

Signal processing & feature extraction

In order to extract appropriate features of the window, we used statistical features from temporal and spectral domains. The temporal features were calculated from the filtered force signals and the spectral features from a single-sided spectrum of the FFT. The left and right features were calculated separately except for correlation coefficient and signal magnitude area between them. These features were then scaled and normalized over the whole feature vectors. Each feature vector was labeled as a value ranged from 1 to 6 depending on its set.

Classification

The first stage of classification was to define a classifier model by finding the best parameters for a given problem. The SVM with RBF kernel has two model parameters: the penalty parameter C ; and the kernel parameter γ. A pairs of (C, γ) of the best accuracy was obtained from both coarse and fine grid-search procedure as shown in Figure 3. A 10-fold leave-one-out cross-validation procedure was also adopted to prevent an overfitting problem. It used nine subjects for training the classifier, while the remaining subject leaved for testing the performance of the classifier. This training-and-testing procedure repeated 10-times by changing the test subject. To evaluate the performance of the classifier, we compared the predicted classes with the labels assigned to each window of the test set. The results for all behaviors were given in Table 1 as a confusion matrix form.

Supplementary Table S1 Extracted features of the time and frequency domain of a window

| # | Feature | # | Feature |
| --- | --- | --- | --- |
| 1 | Standard deviation | 17 | Prediction ratio |
| 2 | Coefficient of variation | 18 | Harmonic ratio |
| 3 | Zero crossing rate | 19 | Fundamental frequency |
| 4 | Percentiles | 20 | Correlation coefficient |
| 5 | Square sum of Percentiles | 21 | Signal magnitude area |
| 6 | Interquartile range | 22 | Spectral energy |
| 7 | Binned distribution | 23 | Spectral zones of energy |
| 8 | Skewness | 24 | Spectral centroid |
| 9 | Kurtosis | 25 | Spectral spread |
| 10 | The 1st Autocorrelation function coefficient | 26 | Spectral entropy |
| 11 | Signal energy | 27 | Spectral entropy of energy |
| 12 | Log signal energy | 28 | Spectral flux |
| 13 | Entropy of energy | 29 | Spectral roll-off |
| 14 | Peak-to-peak amplitude | 30 | Spectral crest |
| 15 | The number of peaks | 31 | Spectral skewness |
| 16 | Time between peaks | 32 | Spectral kurtosis |

1. ^†^ These authors equally contributed to this work as corresponding authors.

   Drs. Won Gu Lee ([termylee@khu.ac.kr](mailto:termylee@khu.ac.kr)) and Hyunwoo Bang ([savoy@snu.ac.kr](mailto:savoy@snu.ac.kr)) [↑](#footnote-ref-2)
